# Lifetime Prediction of Permanent Magnet Synchronous Motor in Selective Compliance Assembly Robot Arm Considering Insulation Thermal Aging

**DOI:** 10.3390/s24123747

**Published:** 2024-06-09

**Authors:** Mingxu Chen, Bingye Zhang, Haibo Li, Xiang Gao, Jiajin Wang, Jian Zhang

**Affiliations:** 1 State Grid Taizhou Power Company, Taizhou 318000, China; 2 College of Electrical Engineering, Zhejiang University, Hangzhou 310011, China

**Keywords:** SCARA, PMSM, insulation thermal aging, lifetime prediction

## Abstract

The direct-drive selective compliance assembly robot arm (SCARA) is widely used in high-end manufacturing fields, as it omits the mechanical transmission structures and has the advantages of high positioning accuracy and fast movement speed. However, due to the intensifying dynamic coupling problem of structures in the direct-drive SCARA, the permanent magnet synchronous motors (PMSMs) located at the joints will take on nonstationary loads, which causes excessive internal temperature and reduces the lifetime of PMSMs. To address these issues, the lifetime prediction of PMSMs is studied. The kinematic and dynamic models of the SCARA are established to calculate the torque curve required by the PMSM in specific typical motion tasks. Additionally, considering thermal stress as the main factor affecting lifetime, accelerated degradation tests are conducted on insulation material. Then, the reliability function of the PMSM is formulated based on the accelerated degradation model. Based on the parameters and working conditions of the PMSM, the temperature field distribution is obtained through simulation. The maximum temperature is used as the reference temperature to conduct reliability evaluation and lifetime prediction of the PMSM. The research results show that for a typical point-to-point task, the PMSM can run for 102,623 h while achieving the reliability requirement of 0.99.

## 1. Introduction

With the rapid development of industrial automation technology, industrial robots have become one of the important means to improve production efficiency, reduce costs, and optimize product quality [1,2]. Industrial robots can perform a variety of heavy, repetitive, and precise tasks, such as assembly, welding, painting, handling, etc., greatly enhancing the flexibility and automation level of production lines [3]. Among various industrial robots, the selective compliance assembly robot arm (SCARA) is widely used due to its simple structure, small footprint, fast movement speed, and high positioning accuracy. It is particularly employed in the semiconductor manufacturing industry to execute point-to-point tasks that require strict operation accuracy [4,5].

SCARA can be divided into two categories based on the driving method: direct-drive SCARA and indirect-drive SCARA. The servo motion system in the indirect-drive SCARA relies on the use of permanent magnet synchronous motors (PMSMs) combined with reducers or mechanical transmission mechanisms like screw rods for high-precision position control [6]. However, inherent mechanical issues in the transmission mechanism, such as gear meshing, clearance, and friction, significantly decrease the positioning accuracy of motion control [7]. Moreover, the overall reliability of the motion system is reduced due to the cumulative impact of fatigue damage on the mechanical transmission mechanisms. Contrastingly, the direct-drive SCARA directly connects the load to the motor, eliminating the intermediary mechanical transmission mechanism, thus avoiding the abovementioned mechanical issues [8]. While the direct-drive SCARA boasts advantages such as compact design, no transmission gap, and high positioning accuracy, it also loses the decoupling characteristics of the mechanical transmission structure. It intensifies the dynamics coupling issues among the various structures, leading to complex drive current waveforms during motion and presenting irregular output torque characteristics. The unsteady torque results in motor overheating, which severely affects the lifetime of the motor.

The key factor limiting the lifetime of a motor is its insulation system. Statistics show that approximately 30% of motor failures are related to the winding insulation [9]. For a low-voltage PMSM used in an SCARA, local discharges are not allowed during operation, so thermal stress is the main aging mechanism affecting the insulation structure system of the motor [10]. When the motor operates under overheating conditions, the heat expansion of the insulation material produces defects, easily leading to insulation breakdowns and causing motor interruption [11]. According to the Arrhenius law, for every 8 to 10 °C rise in temperature, the insulation lifetime will be halved [12]. While effective thermal management of the motor can be achieved through methods like optimizing cooling design and providing effective cooling approaches, these measures are not conducive to the lightweight and refined design of robotic arms. Therefore, establishing a rational reliability assessment model is a more effective method. Carrying out reliability assessments and lifetime prediction of the motor insulation system can effectively improve the operating efficiency of the motor, reducing the economic loss and maintenance costs caused by equipment failures.

Traditional reliability assessment techniques aim to obtain failure data and are often plagued by extensive test cycles and high costs. Especially for long-lifetime, high-reliability products such as motor insulation systems, even under more severe stress levels to accelerate the degradation of insulation, it is challenging to gather sufficient failure data to establish a reliability model [13]. However, the failure of the insulation system is generally associated with several dielectric parameters such as insulation resistance, insulation capacitance, breakdown voltage, and dielectric loss factor. These parameters can all be used to characterize the degree of insulation aging [14,15,16]. Therefore, reliability assessment models based on insulation aging diagnostic parameters have received extensive attention. By collecting accelerated degradation data from tests carried out at stress levels higher than normal operating levels, the degradation trajectory of the product is captured using curve fitting [17], stochastic processes [18], neural networks [19], etc., and product lifetime is predicted based on pre-=set thresholds. Due to its excellent computational properties and strong interpretability, the Wiener process is particularly suited to the degradation process of motor insulation structures under thermal stress, thus improving the precision of reliability assessment [18].

Aiming at the issue of internal temperature rise in PMSMs due to complex drive currents and irregular output torque when executing typical point-to-point tasks in direct-drive SCARA robotic arms [20], a reliability assessment model under thermal stress is proposed. Firstly, kinematic and dynamic models of the direct-drive SCARA are established, and from the general trajectory planning method, the motion curve to complete a given motion task is obtained and the required torque for the motor is derived. Since thermal stress is identified as the main aging stress for the insulation system of low-voltage PMSM, accelerated thermo-aging tests were conducted on typical insulation materials. A reliability assessment model is established by modeling the degradation path based on the stochastic process model and the Arrhenius model. Finally, a temperature field analysis of the motor is performed and the highest temperature is taken as the reference temperature for the reliability assessment of the motor.

The main content of this paper is as follows. Section 2 establishes the kinematics and dynamics models of the SCARA. Section 3 designs and conducts accelerated thermal aging tests on insulation structures, and a motor insulation reliability model under thermal stress is established based on the Wiener process. Section 4 conducts a case study on a typical SCARA operating condition, analyzing its output torque, temperature field, and reliability. Section 5 summarizes the entire article.

## 2. The Kinematic and Dynamic Models of SCARA

The kinematic and dynamic models of the SCARA are crucial for analyzing its movement patterns and are vital in design, control, and performance evaluation. The inverse kinematic analysis of the SCARA is carried out to accurately calculate the joint angles of the robotic arm, thereby realizing the expected position and attitude of the end effector. Moreover, in order to describe the relationship between the motor torque and the movement of the robotic arm, a dynamic model of the SCARA is established.

### 2.1. Kinematic Model

**Figure 1:**
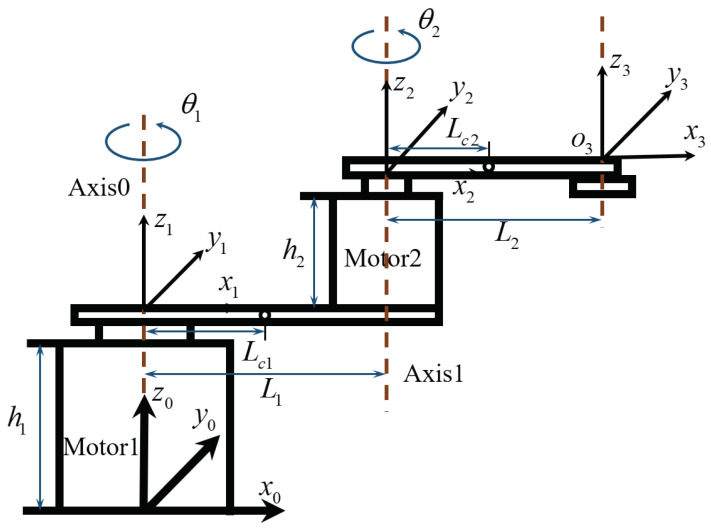
The schematic diagram of the direct-drive SCARA.

The kinematics of the SCARA robotic arm are described by the relationship between the joint variables and the coordinates of the end effector in the Cartesian coordinate system without considering the forces and torque that cause movement. According to the structure of the directly driven SCARA under study, a corresponding kinematic model and reference frame are established. As shown in Figure 1, its structure mainly includes two direct-drive motors, two connecting rods, and one equivalent load. The reference coordinate system of SCARA is established based on the Denavit–Hartenberg (DH) convention.

The DH parameters of the direct-drive SCARA are shown in Table 1. $L_{1}$ and $L_{2}$ represent the lengths of links 1 and 2, respectively. The rotation angles of joint 1 and joint 2 are denoted by $\theta_{1}$ and $\theta_{2}$, respectively. $h_{1}$ and $h_{2}$ signify the heights of motor 1 and motor 2, respectively.

Under the DH convention, based on the coordinate transformation relationship, we obtain the replacement matrix corresponding to the link transformation. The homogeneous transformation matrix, which characterizes the position and posture of adjacent coordinate systems $x_{i}y_{i}z_{i}$ relative to $x_{j}y_{j}z_{j}$, is as follows:

(1) $${\;}_{i}^{j}T=\left[\begin{array}{cccc} \cos\theta_{i} & -\sin\theta_{i} & 0 & x_{i}\\ \sin\theta_{i}\cos\alpha_{j} & \cos\theta_{i}\cos\alpha_{j} & -\sin\alpha_{j} & y_{i}\\ \sin\theta_{i}\sin\alpha_{j} & \cos\theta_{i}\sin\alpha_{j} & \cos\alpha_{j} & z_{i}\\ 0 & 0 & 0 & 1 \end{array}\right],$$

where $x_{i}=a_{i-1}$, $y_{i}=-d_{i}\sin\alpha_{j}$, $z_{i}=d_{i}\cos\alpha_{j}$, j=i−1, i=1,2,3.

By substituting the model parameters of the SCARA into Equation (1), the coordinate transformation relationship between adjacent joints in the SCARA can be obtained:

(2) $${\;}_{1}^{0}T=\left[\begin{array}{cccc} \cos\theta_{1} & -\sin\theta_{1} & 0 & 0\\ \sin\theta_{1} & \cos\theta_{1} & 0 & 0\\ 0 & 0 & 1 & h_{1}\\ 0 & 0 & 0 & 1 \end{array}\right],$$

(3) $${\;}_{2}^{1}T=\left[\begin{array}{cccc} \cos\theta_{2} & -\sin\theta_{2} & 0 & L_{1}\\ \sin\theta_{2} & \cos\theta_{2} & 0 & 0\\ 0 & 0 & 1 & h_{2}\\ 0 & 0 & 0 & 1 \end{array}\right],$$

(4) $${\;}_{3}^{2}T=\left[\begin{array}{cccc} 1 & 0 & 0 & L_{2}\\ 0 & 1 & 0 & 0\\ 0 & 0 & 1 & 0\\ 0 & 0 & 0 & 1 \end{array}\right].$$

The homogeneous transformation matrix of the end of the SCARA relative to the inertial coordinate system $x_{0}y_{0}z_{0}$ established on the base of motor 1 is

(5) $${\;}_{3}^{0}T={\;}_{1}^{0}T\cdot {\;}_{2}^{1}T\cdot {\;}_{3}^{2}T=\left[\begin{array}{cccc} \cos\left(\theta_{1}+\theta_{2}\right) & -\sin\left(\theta_{1}+\theta_{2}\right) & 0 & x\\ \sin\left(\theta_{1}+\theta_{2}\right) & \cos\left(\theta_{1}+\theta_{2}\right) & 0 & y\\ 0 & 0 & 1 & z\\ 0 & 0 & 0 & 1 \end{array}\right],$$

where $x=L_{1}\cos\theta_{1}+L_{2}\cos\left(\theta_{1}+\theta_{2}\right)$, $y=L_{1}\sin\theta_{1}+L_{2}\sin\left(\theta_{1}+\theta_{2}\right)$, and $z=h_{1}+h_{2}$.

**Figure 2:**
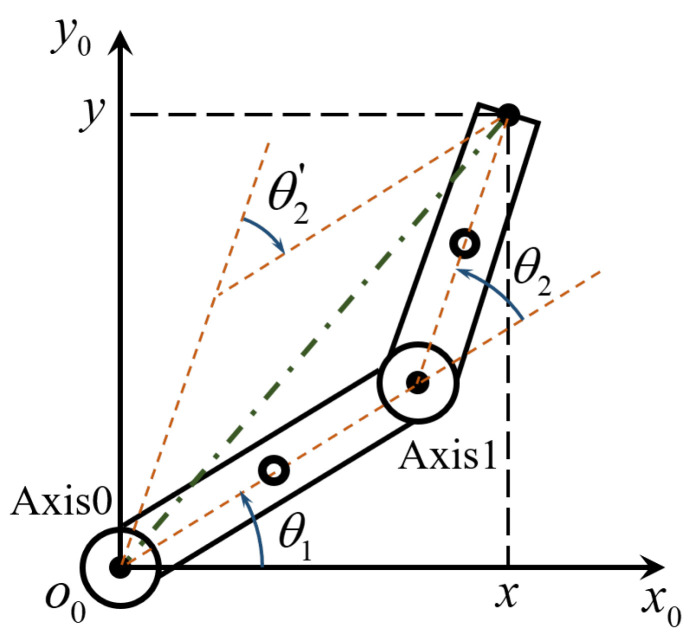
The schematic diagram of the inverse motion structure of the direct-drive SCARA.

The inverse kinematics solution of the SCARA is to obtain the angle values of each link in the joint space, i.e., $\theta_{1}$ and $\theta_{2}$, based on the coordinates (x,y,z) of the end effector in the Cartesian space under the condition of knowing the geometric parameters of each link. Since the studied direct-drive SCARA does not move along the *z*-axis, its equivalent motion model in the $o_{0}x_{0}y_{0}$ plane is shown in Figure 2.

The inverse kinematics solution for SCARA is obtained using algebraic methods. According to the cosine theorem, $\theta_{2}$ can be expressed as

(6) $$\theta_{2}=\arccos\frac{x^{2}+y^{2}-L_{1}^{2}-L_{2}^{2}}{2L_{1}L_{2}}.$$

As can be seen from Figure 2, the same end coordinate can yield two different postures, i.e., ${\theta_{2}}^{\prime }=-\theta_{2}$. According to the method in [21], after variable substitution, $\theta_{1}$ can be expressed as

(7) $$\theta_{1}=\arctan\frac{y}{x}-\arctan\frac{k_{2}}{k_{1}},$$

where $k_{1}=L_{1}+L_{2}\cos\theta_{2}$ and $k_{2}=L_{2}\sin\theta_{2}$.

### 2.2. Dynamic Model

The dynamic equations of the SCARA can effectively clarify the relationship between force or torque and motion. According to the basic structural parameters of the SCARA in Figure 1, the Euler–Lagrange equation is used to establish the dynamic model. Since the movement of the investigated SCARA is limited to the horizontal plane, gravitational potential energy is not considered.

In order to establish the dynamic model, the inertia matrix needs to be derived first, which can be expressed as

(8) $$M\left(\theta_{1},\theta_{2}\right)=\left[\begin{array}{cc} M_{11} & M_{12}\\ M_{21} & M_{22} \end{array}\right],$$

where $M_{11}=m_{1}L_{c1}^{2}+m_{2}\left(L_{1}^{2}+L_{c2}^{2}+2L_{1}L_{c2}\cos\theta_{2}\right)+I_{1}+I_{2}$, $M_{12}=M_{21}=m_{2}\left(L_{c2}^{2}+\right.$ $\left.L_{1}L_{c2}\cos\theta_{2}\right)+I_{2}$, and $M_{22}=m_{2}L_{c2}^{2}+I_{2}$. $m_{1}$ and $m_{2}$, respectively, represent the mass of link 1 and link 2, $L_{c1}$ and $L_{c2}$, respectively, represent the distance between the previous joint and the centroid of link 1 and link 2, and $I_{1}$ and $I_{2}$, respectively, represent the moment of inertia of link 1 and link 2 about the *z*-axis passing through their centroid.

According to [22], the Christoffel symbols are expressed as $c_{111}=c_{122}=c_{212}=c_{222}=0$, $c_{121}=c_{211}=c_{221}=c_{112}=-m_{2}L_{1}L_{c2}\sin\theta_{2}$.

The torque generated by the motor at the joint can be divided into three parts: the torque $\tau_{mi}$ required for motor rotation, the torque $\tau_{fi}$ required to overcome friction, and the disturbance torque $\tau_{di}$ generated by dynamic coupling. The torque $\tau_{mi}$ can be expressed as

(9) $$\tau_{mi}=\left(J_{i}+\frac{M_{ii}}{N_{i}^{2}}\right){\ddot{q}}_{i},$$

where $q_{i}$ represents the rotation angle of the motor; $q_{i}=N_{i}\theta_{i}$, $N_{i}$ is the reduction ratio, $J_{i}$ is the moment of inertia of the motor itself, and ${\ddot{q}}_{i}$ is the second derivative of $q_{i}$ with respect to time, i=1,2.

Only considering the friction at the joint, based on the Coulomb and viscous friction model, $\tau_{fi}$ can be calculated and expressed as

(10) $$\tau_{fi}=B_{vi}{\dot{q}}_{i}+F_{ci}\mathrm{sign}\left({\dot{q}}_{i}\right),$$

where $B_{vi}$ represents the viscous friction coefficient, $F_{ci}$ is the Coulomb friction coefficient, and ${\dot{q}}_{i}$ represents the first derivative of $q_{i}$ with respect to time.

According to the Euler–Lagrange equation, the disturbance torques $\tau_{d1}$ and $\tau_{d2}$ generated by the dynamic coupling of two motors can be respectively represented as

(11) $$\tau_{d1}=M_{12}{\ddot{\theta }}_{2}+\left(c_{121}+c_{211}\right){\dot{\theta }}_{1}{\dot{\theta }}_{2}+c_{221}{\dot{\theta }}_{2}^{2},$$

(12) $$\tau_{d2}=M_{21}{\ddot{\theta }}_{1}+c_{112}{\dot{\theta }}_{1}^{2}.$$

Therefore, the output torques $\tau_{1}$ and $\tau_{2}$ of the motor can be derived as

(13) $$\begin{array}{cc} \tau_{1} & =\tau_{m1}+\tau_{f1}+\tau_{d1}\\ \; & =\left(J_{1}+\frac{M_{11}}{N_{1}^{2}}\right)N_{1}{\ddot{\theta }}_{1}+B_{v1}N_{1}{\dot{\theta }}_{1}+F_{c1}\mathrm{sign}\left(N_{1}{\dot{\theta }}_{1}\right)\\ \; & +\frac{1}{N_{1}}\left[M_{12}{\ddot{\theta }}_{2}+\left(c_{121}+c_{211}\right){\dot{\theta }}_{1}{\dot{\theta }}_{2}+c_{221}{\dot{\theta }}_{2}^{2}\right], \end{array}$$

(14) $$\begin{array}{cc} \tau_{2} & =\tau_{m2}+\tau_{f2}+\tau_{d2}\\ \; & =\left(J_{2}+\frac{M_{22}}{N_{2}^{2}}\right)N_{2}{\ddot{\theta }}_{2}+B_{v2}N_{2}{\dot{\theta }}_{2}+F_{c2}\mathrm{sign}\left(N_{2}{\dot{\theta }}_{2}\right)\\ \; & +\frac{1}{N_{2}}\left(M_{21}{\ddot{\theta }}_{1}+c_{112}{\dot{\theta }}_{1}^{2}\right). \end{array}$$

For a direct-drive SCARA, $N_{1}=N_{2}=1$.

## 3. Reliability Model of Motor under Thermal Stress

The aging process of motor insulation structures has a certain degree of randomness, which is suitable for degradation modeling using the Wiener process [23]. Degradation processes under different thermal stresses follow the same random process, but their model parameters are different. By combining with the Arrhenius equation, model parameters under normal thermal stress can be extrapolated. After setting the failure threshold, based on the concept of first passage time, the distribution function of the failure time of the motor insulation structure can be determined from the degradation model, thus achieving the reliability assessment of the motor.

### 3.1. Degradation Model Based on Wiener Process

Assuming that the performance degradation process of the insulation structure follows a linear Wiener process, let Y(t) represent the amount of insulation diagnostic performance degradation at time *t*. Based on the Wiener process, the degradation model is represented as

(15) $$Y\left(t\right)=\mu t+\sigma B\left(t\right)+y\left(0\right),$$

where μ is the drift parameter of the Wiener process, σ is the diffusion parameter, B(·) is the standard Brownian motion, and y(0) is the initial amount of degradation.

For high-reliability and long-lifetime products such as motor insulation structures, a degradation model is usually established by conducting multiple sets of accelerated degradation tests to collect sufficient accelerated degradation data. When establishing a Wiener process degradation model based on accelerated degradation data, the relationship between the model parameters and the stress needs to be determined first. Ref. [24] proved that both the drift parameter and the diffusion parameter in the Wiener process are related to the accelerated stress level. According to the principle of accelerated factor consistency, the accelerated factor $A_{k,h}$ is a constant determined only by the stress levels $S_{k}$ and $S_{h}$. In the Wiener process, the relationship between $A_{k,h}$ and μ, σ can be expressed as

(16) Ak,h=μkμkμhμh=σk2σk2σh2σh2.

To establish this relationship, an acceleration model is usually used to describe the pattern between degradation model parameters and stress levels. Thermal stress is the main factor causing motor insulation deterioration, and the thermal aging mechanism of insulation materials is internal chemical reaction. The Arrhenius model is suitable for describing the aging rate as a function of temperature. The reaction rate equation for thermal effect is

(17) $$\zeta =M\exp\left(\frac{E_{a}}{\kappa T}\right),$$

where ζ represents the reaction rate, which usually represents the degradation model parameters affected by the stress level. *T* represents the thermal stress level, $E_{a}$ represents the material’s activation energy, and *M* and κ are related parameters. From Equation (17), it can be seen that the higher the stress level, the faster the reaction rate, and the larger the degradation model parameters of the insulation structure. Therefore, the drift parameter under the *k*-th thermal stress $T_{k}$ is expressed as

(18) $$\mu_{k}=\exp\left(\gamma_{1}-\gamma_{2}/T_{k}\right).$$

The diffusion parameter under the *k*-th thermal stress $T_{k}$ is expressed as

(19) $$\sigma_{k}=\exp\left(\gamma_{3}-\gamma_{4}/T_{k}\right),$$

where $\gamma_{1},\gamma_{2},\gamma_{3},\gamma_{4}$ are coefficients to be determined. To satisfy Equation (16), $\gamma_{4}=0.5\gamma_{2}$. The accelerated degradation model is established as

(20) $$Y\left(t\right)\left(\exp\left(\gamma_{1}-\gamma_{2}/T\right)t,\exp\left(2\gamma_{3}-\gamma_{2}/T\right)t\right).$$

Based on the independent increment characteristics of the Wiener process, the following likelihood function is established:

(21) $$L\left(\gamma_{1},\gamma_{2},\gamma_{3}\right)=\prod_{k=1}^{N}\prod_{j=1}^{N_{k}}\prod_{i=1}^{N_{jk}}\frac{1}{\sqrt{2\pi \sigma_{k}^{2}\Delta t_{ijk}}}\exp\left[-\frac{{\left(\Delta y_{ijk}-\exp\left(\gamma_{1}-\gamma_{2}/T_{k}\right)\Delta t_{ijk}\right)}^{2}}{2\exp\left(2\gamma_{3}-\gamma_{2}/T_{k}\right)\Delta t_{ijk}}\right],$$

where $y_{ijk}$ is the degradation value of performance measured for the *i*-th time on the *j*-th product under the *k*-th stress level, $t_{ijk}$ is the corresponding measurement time, $\Delta y_{ijk}=y_{ijk}-y_{(i-1)jk}$ is the degradation increment, and $\Delta t_{ijk}=t_{ijk}-t_{(i-1)jk}$ is the time increment. $N_{jk}$ is the number of times the *j*-th sample was measured under the *k*-th stress level, $N_{k}$ is the number of samples under the *k*-th stress level, and *N* is the number of accelerated stresses.

By utilizing the maximum likelihood estimation method, one can solve for $\gamma_{1},\gamma_{2},\gamma_{3}$, and, thus, predict the degradation model of motor insulation under normal working stress levels.

### 3.2. Accelerated Thermal Aging Test and Data Analysis

**Figure 3:**
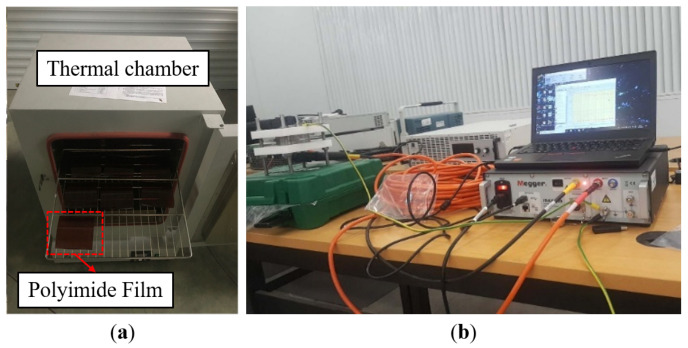
Test platform: (**a**) Insulation aging platform; (**b**) Insulation diagnostic system.

In order to study the impact of thermal stress on the insulation performance of motors, aging tests were conducted with typical motor insulation materials as the research subject. Polyimide film with a heat rating above 200 °C and a thickness of 0.25 mm was selected as the sample, as shown in Figure 3a. A total of 21 samples were produced from the same batch of products, which were randomly divided into three groups for accelerated aging tests. According to the requirements of IEC 60505 [25], in order to cause rapid aging of the samples and ensure consistency between the failure mechanisms under accelerated stress and working stress, three constant accelerated stress levels were chosen: $T_{1}=563.15$ K, $T_{2}=573.15$ K, and $T_{3}=583.15$ K. Seven samples were placed into the oven shown in Figure 3a for thermal aging under each group of accelerated thermal stress. Before the accelerated aging test, each sample was preprocessed and its insulation performance characterization parameters were measured at its unaged state, with this value serving as the initial value. The aging cycle under three different temperatures was set as shown in Table 2. After each aging cycle, insulation diagnostic tests were conducted on the samples. In these tests, the Megger IDX300 Insulation Diagnostic System was used for insulation performance testing, as shown in Figure 3b.

**Figure 4:**
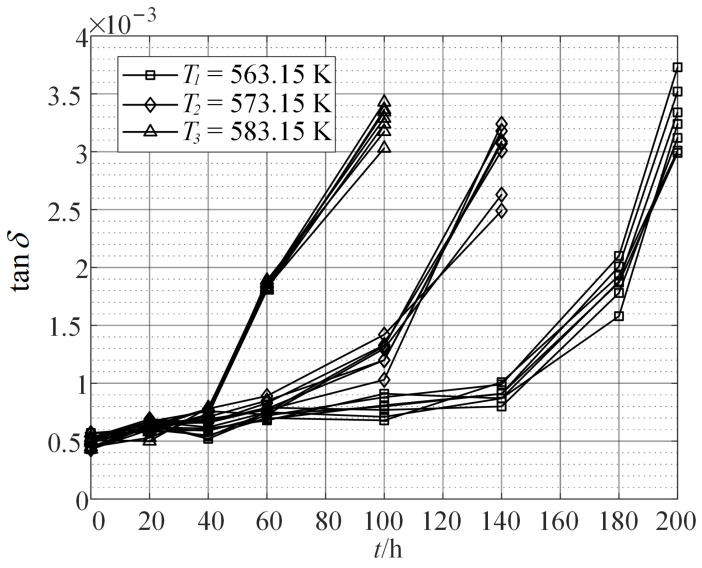
Accelerated degradation data of insulation materials.

The tangent of the dielectric loss angle, tanδ, is selected as the indicator to characterize performance degradation. The accelerated aging data are shown in Figure 4. The higher the temperature, the faster the rate of performance degradation.

By fusing all the accelerated degradation data and establishing the maximum likelihood function according to Equation (21), the maximum likelihood estimation values of the model parameters $\left(\gamma_{1},\gamma_{2},\gamma_{3}\right)=\left(6.28,9831.64,-0.74\right)$ are obtained. By substituting the estimated values of $\gamma_{1},\gamma_{2},\gamma_{3}$ into Equations (18) and (19), the relationship between the degradation model parameters based on the Wiener process and the thermal stress level is obtained as follows:

(22) μT=exp2.92−7904.797904.79TTσT=exp−2.33−3952.393952.39TT.

**Figure 5:**
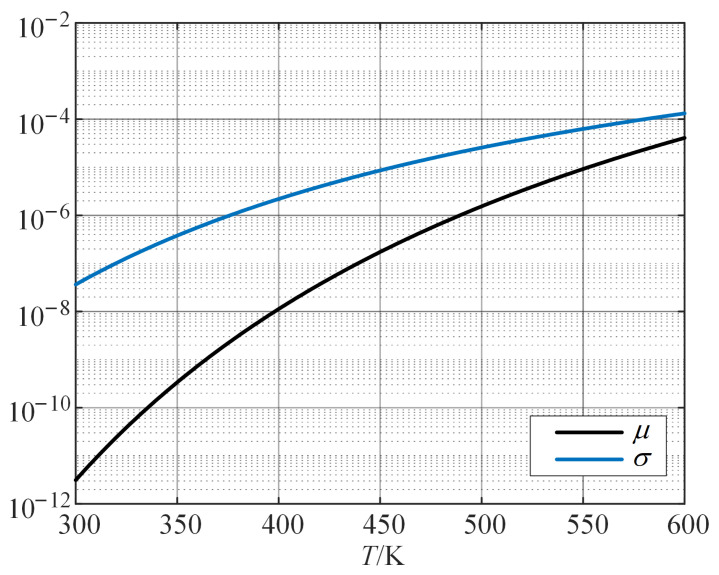
μ, σ curve with thermal stress level.

According to Equation (22), the relationship between μ,σ in the Wiener process and the thermal stress level is plotted as shown in Figure 5. The higher the level of thermal stress, the larger the values of μ and σ. The degradation model established based on the accelerated degradation data can be extrapolated to the degradation model of motor insulation under working stress level, and used for reliability evaluation.

### 3.3. Reliability Evaluation Model

Let *D* be the failure threshold of the characteristic parameters of motor insulation performance. Based on the first passage time theory, the lifetime *L* of motor insulation is defined as the time when Y(t) first passes *D*, that is,

(23) $$L=\inf\left\{t|Y(t)\geq D\right\}.$$

Based on the properties of the Wiener process, the lifetime *L* of motor insulation follows an inverse Gaussian distribution. Its probability density function $f_{L}\left(t\right)$ and cumulative distribution function $F_{L}\left(t\right)$ can be expressed as

(24) $$f_{L}\left(t\right)=\frac{D}{\sqrt{2\pi \sigma^{2}t^{3}}}\exp\left[-\frac{{\left(D-\mu t\right)}^{2}}{2\sigma^{2}t}\right],$$

(25) $$F_{L}\left(t\right)=\mathrm{Pr}\left(L\leq t\right)=\Phi \left(\frac{\mu t-D}{\sigma \sqrt{t}}\right)+\exp\left(\frac{2\mu D}{\sigma^{2}}\right)\Phi \left(-\frac{\mu t+D}{\sigma \sqrt{t}}\right),$$

where Φ(·) is the cumulative distribution function of the standard normal distribution. The reliability function $R_{L}\left(t\right)$ can be expressed as

(26) $$R_{L}\left(t\right)=1-F_{L}\left(t\right)=\Phi \left(\frac{D-\mu t}{\sigma \sqrt{t}}\right)-\exp\left(\frac{2\mu D}{\sigma^{2}}\right)\Phi \left(-\frac{\mu t+D}{\sigma \sqrt{t}}\right),$$

According to IEC 60674-2 [26], we set the failure threshold of tan δ to D=0.005. Substituting Equation (22) into Equation (26), the reliability function under any thermal stress level *T* can be obtained as

(27) RLt,T=Φ0.005−exp2.92−7904.797904.79TTtexp−2.33−3952.393952.39TTt−exp0.01exp2.92−7904.797904.79TTexp−4.66−7904.797904.79TTΦ−exp2.92−7904.797904.79TTt+0.005exp−2.33−3952.393952.39TTt.

## 4. Case Study

Firstly, the motion trajectory of the direct-drive SCARA is derived by utilizing the S-curve, a widespread technique in the planning of robot arm motion paths. Based on the kinematic and dynamic models of the SCARA, the output torque of the motor is calculated. The motor employed is a surface-mounted PMSM. With the use of the computational fluid dynamics (CFD) model, the temperature field distribution of the motor under the given operating conditions is determined. Finally, the reliability evaluation of the motor is conducted. The structural parameters of the SCARA are shown in Table 3.

### 4.1. Output Torque Analysis

**Figure 6:**
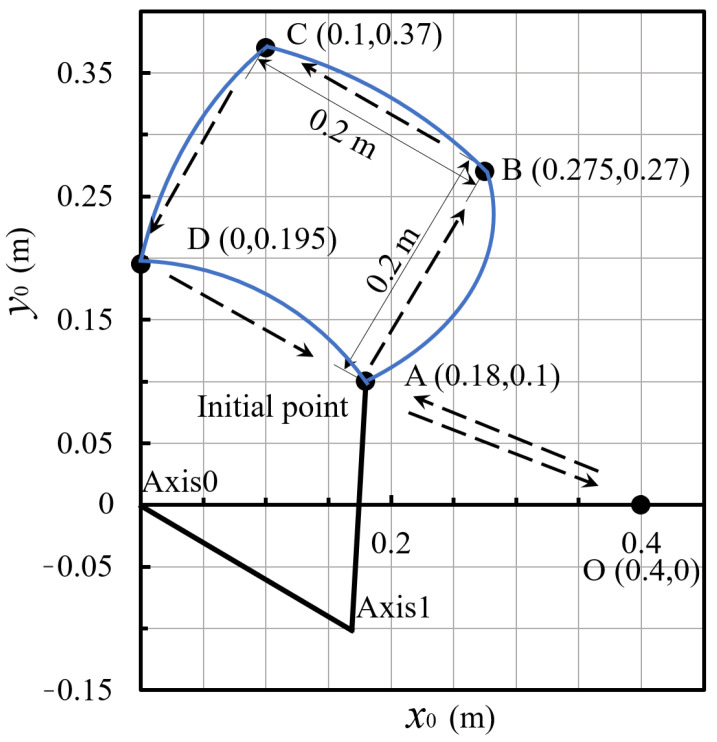
The motion task diagram of the direct-drive SCARA.

In the experiment, the motion task is set as a point-to-point movement within a 200 mm × 200 mm square range. As shown in Figure 6, the points of movement are A, B, C, and D, with a point-to-point movement duration of $t_{m}=400$ ms, and each movement section has a dwell time of $t_{d}=100$ ms. The motion task simulates a typical assembly operation of a direct-drive SCARA in industrial applications. The motion trajectory of the SCARA, obtained using the S-curve, is shown as the blue curve in Figure 6.

**Figure 7:**
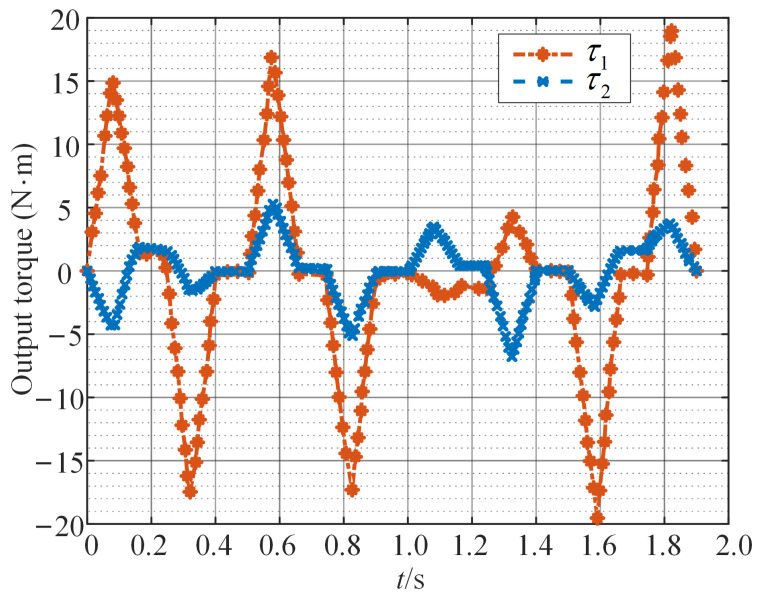
The output torque of motors in the SCARA.

**Figure 8:**
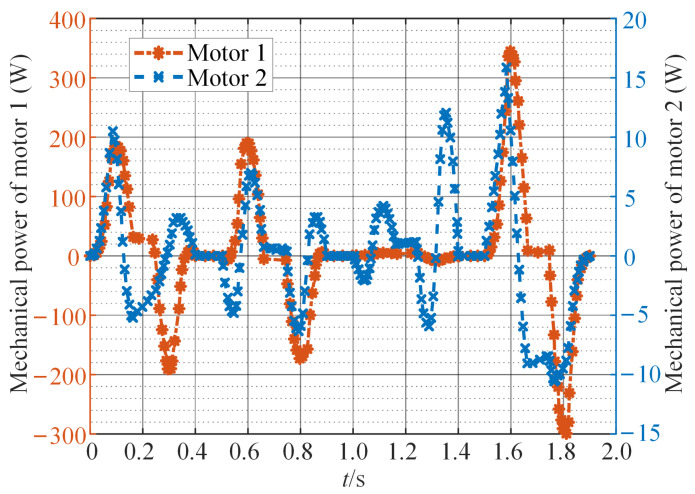
The mechanical power of motors in the SCARA.

**Figure 9:**
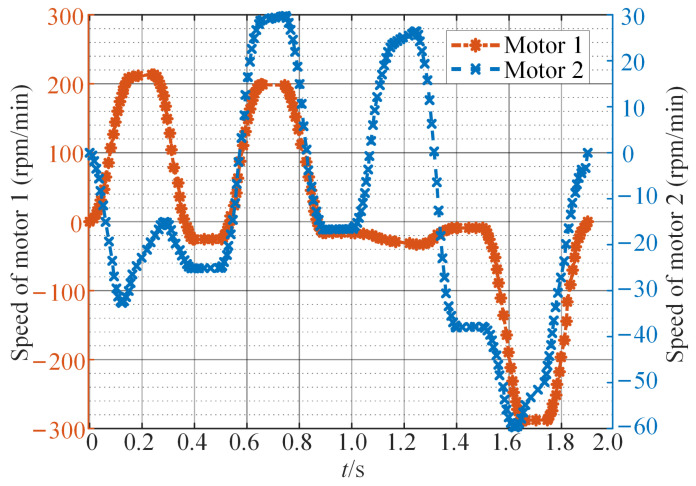
The speed of motors in the SCARA.

For general direct-drive point-to-point servo motion systems, the ratio of load to motor’s rotational inertia is often greater than 20:1. The influence of the motor’s rotational inertia on the continuous torque, peak torque, and power required by the motor is negligible. Therefore, when calculating the output torque of the motor, the motor’s own rotational inertia can be ignored. In Equations (13) and (14), let $J_{1}=J_{2}=0$. Based on the dynamic equations of the SCARA, the output torque of the motor is calculated during the execution of a complete motion task. As shown in Figure 7, motor 1 on axis 0 requires a larger and more irregular output torque to perform this motion task. Additionally, the mechanical power and speed of the motor are provided in Figure 8 and Figure 9. Negative mechanical power indicates that the motor is in braking mode, where the torque and speed directions are opposite, and torque is applied in the reverse direction to slow down or stop the rotation. Due to the strong coupling effect between the two axes of the direct-drive SCARA, when the motors on both axes rotate in the same direction, the motor on axis 0 needs to output higher torque to counteract the influence of axis 1. Conversely, motion in opposite directions beneficially utilizes the coupling effect, requiring less torque from the motor and resulting in more stable movement. The operation of a permanent magnet motor under nonstationary load leads to a significant rise in internal temperature, accelerating the deterioration process of the insulation structure, degrading the motor’s performance, or even causing a failure.

### 4.2. Temperature Field Analysis

**Figure 10:**
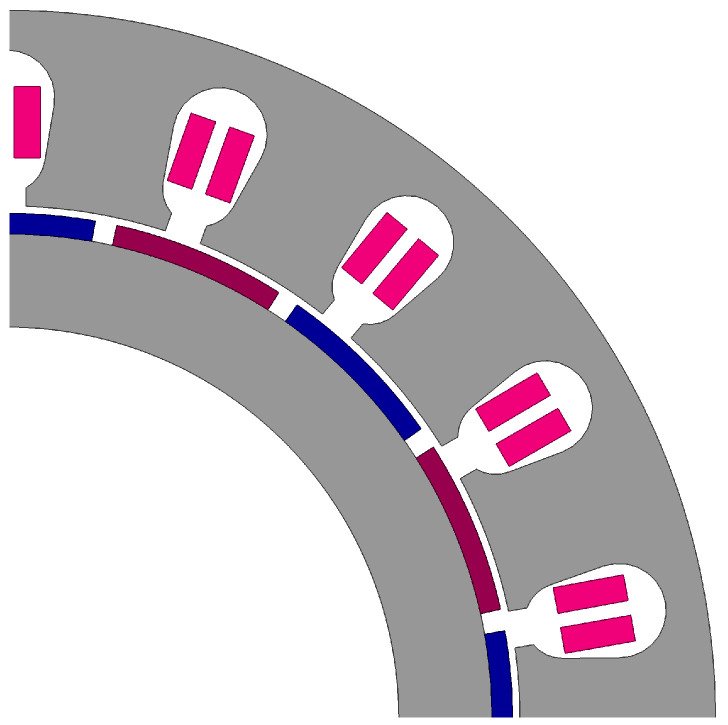
The electromagnetic model of motor 1.

To conduct a conservative reliability evaluation of the direct-drive SCARA, motor 1 is selected as the research object, and its temperature field distribution in an air medium is analyzed. Motor 1 is a PMSM, with its main parameters as shown in Table 4. To determine the current required to achieve the torque curve for motor 1, an electromagnetic finite element simulation model is established based on its structural parameters, as shown in Figure 10. Multiple sets of currents are applied to the windings, and the torque is solved through simulation.Based on the simulation results, a mapping relationship between torque $\tau_{1}$ and current $I_{1}$ is established as follows:

(28) $$\tau_{1}=2.05I_{1}.$$

**Figure 11:**
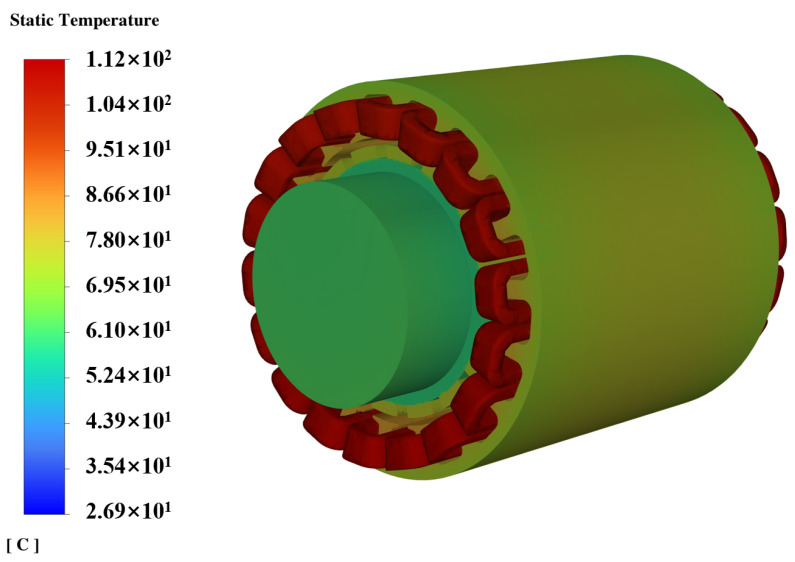
The temperature field distribution of motor 1.

**Figure 12:**
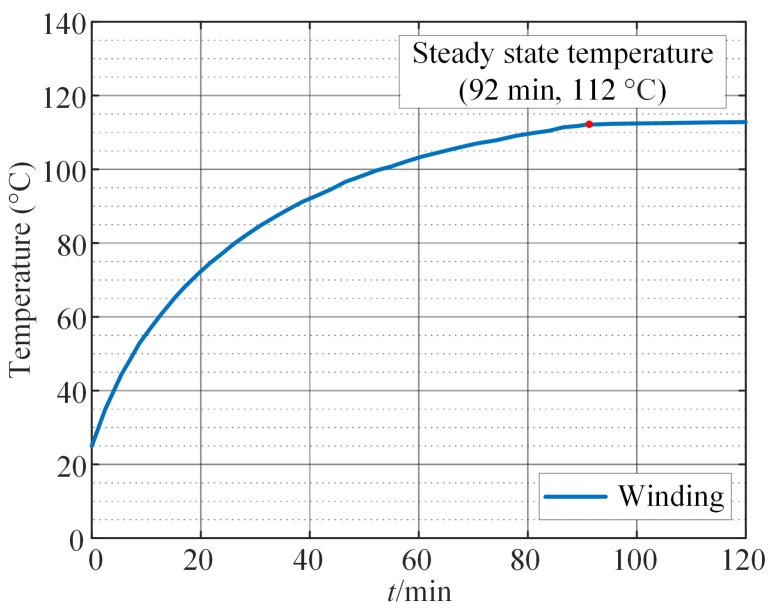
The transient temperature rise curve of winding of motor 1.

Since the motor operates at low speeds while performing the task, iron loss can be neglected in the temperature field simulation, considering only copper loss as the heat source. The current of the motor varies throughout the working cycle. From a thermodynamic perspective and based on the principle of constant heat generation, copper loss is calculated using the average equivalent current. Due to the rotation of the motor rotor, permanent magnets, and other structures, the internal airflow forms vortices, and is mainly turbulent, so a turbulence model is adopted. In the CFD thermal simulation, gravity is activated, and air density is set to vary with temperature to simulate natural convection around the motor. The RNG k−ϵ model is used for turbulence modeling. When setting the heat source, the impact of temperature on copper loss is considered, and the UDF method is used to realize the magnetic–thermal coupling. Meanwhile, the following settings are made for the boundary conditions: the solid and fluid parts are coupled through an interface, and the contact surface of the rotating parts and the fluid is set as a rotating wall without relative sliding. The external boundary of the air domain around the motor is set as a pressure outlet with fixed temperature wall conditions. Based on the CFD method, the temperature field distribution of motor 1 is simulated as shown in Figure 11. As can be seen from Figure 11, the worst part under thermal stress internally in the motor is the stator winding, with its end reaching 112 °C, and its transient temperature rise curve is shown in Figure 12. The highest thermal stress level is considered the worst operating condition for the motor and is used for reliability analysis.

### 4.3. Reliability Analysis

By substituting the thermal stress level T=385.15 K into Equation (27), the reliability function of motor 1 can be expressed as

(29) $$R_{L}\left(t\right)=\Phi \left(\frac{0.005-2.26\times 10^{-8}t}{3.41\times 10^{-6}\sqrt{t}}\right)-2.84\times 10^{8}\Phi \left(-\frac{2.26\times 10^{-8}t+0.005}{3.41\times 10^{-6}\sqrt{t}}\right).$$

**Figure 13:**
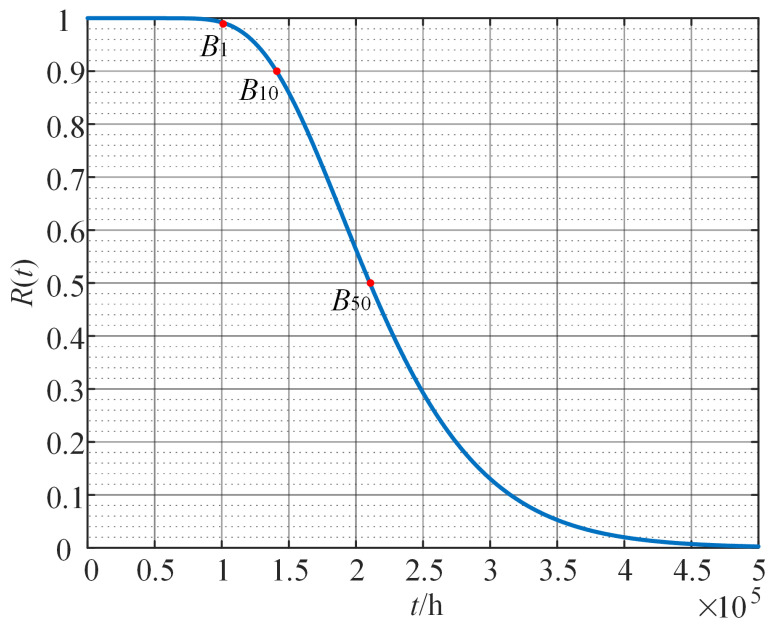
The reliability curve of motor 1.

The reliability curve of motor 1 is shown in Figure 13. Based on this, given the expected reliability threshold λ, that is, $\lambda =R_{L}\left(t_{\lambda }\right)$, the thermal lifetime $t_{\lambda }$ of motor 1 can be obtained by $t_{\lambda }=R_{L}^{-1}\left(\lambda \right)$, where $R_{L}^{-1}\left(\cdot \right)$ represents the inverse function of the reliability function in Equation (29). The lifetime percentile $B_{\rho }\left(\rho =100\left(1-\lambda \right)\right)$ is also given by the definition of $t_{\lambda }$, which indicates that ρ% of the samples will reach the end of life at time $t_{\lambda }$. For example, when λ is set to 0.99, $t_{0.99}$ is referred to as $B_{1}$ lifetime, that is, when the time reaches $t_{0.99}$, 1% of the samples fail. Table 5 presents several typical life percentiles.

## 5. Conclusions

For the typical movement tasks of a direct-drive SCARA, the operating condition of the PMSM during the task is investigated, and an evaluation method for the motor reliability considering thermal stress as the main aging factor is proposed. By establishing the kinematics and dynamics models for the SCARA, the output torque curve required by the motor is calculated. Accelerated thermal aging tests are conducted for insulation material, and an accelerated degradation model is established for the aging data based on the Wiener process and Arrhenius equation. From this, the reliability function of the motor is derived. Using the CFD method, the temperature field distribution of the motor is simulated based on its structure and operating condition. With the highest temperature as the reference, the motor reliability is analyzed. The research results show that, in a typical point-to-point task of SCARA, the motor can operate for 102,623 h continuously under a reliability requirement of 99%.

The reliability evaluation of a motor can enrich the understanding of its long-term performance and provide a crucial reference for improving the overall reliability of the robotic arm, thus reliably supporting industrial automated production. However, there are still some issues that deserve further research. The multistress lifetime models can be developed to achieve more comprehensive lifetime prediction for practical applications by considering the effects of electrical, mechanical, and environmental aging on insulation. In addition, if the motor is subjected to thermal shocks caused by temperature jumps during operation, it is more appropriate to establish a degradation model considering the shock process to describe the insulation aging process, which can reach a more accurate lifetime prediction. The lifetime prediction of electrical machines in the scheduled operation is also an interesting topic for further research.

## Figures and Tables

**Table 1:** The DH parameters of the direct-drive SCARA; $a_{i-1}$, $\alpha_{i-1}$, $d_{i}$, and $\theta_{i}$ are, respectively, the length of link i−1, torsion angle of link i−1, offset of joint *i*, and joint angle of joint *i*.

Joint *i*	$a_{i-1}$	$\alpha_{i-1}$	$d_{i}$	$\theta_{i}$
1	0	0	$h_{1}$	$\theta_{1}$
2	$L_{1}$	0	$h_{2}$	$\theta_{2}$
3	$L_{2}$	0	0	0

**Table 2:** Aging period at different thermal stress levels.

Thermal Stress Level	Aging Time/h						
$T_{1}$	20	40	60	100	140	180	200
$T_{2}$	20	40	60	100	140		
$T_{3}$	20	40	60	100			

**Table 3:** The structural parameters of the direct-drive SCARA.

Parameters	Unit	Value
$L_{1}$	m	0.2
$L_{2}$	m	0.2
$L_{c1}$	m	0.095
$L_{c2}$	m	0.091
$m_{1}$	kg	1.55
$m_{2}$	kg	0.73

**Table 4:** The key parameters of motor 1.

Parameters	Unit	Value
Number of stator slots		18
Number of pole pairs		8
Winding configuration		Centralization
Rated power	W	200
Maximum speed	rpm/min	500
Maximum torque	N·m	20
Effective length of motor	mm	72
Air gap	mm	0.8
Outer radius of the stator	mm	76
Inner radius of the rotor	mm	42
Outer radius of the rotor	mm	52
Thickness of permanent magnet	mm	2.2
Thickness of tooth-tip	mm	2
Length of tooth	mm	14
Thickness of stator yoke	mm	5
Diameter of conductor	mm	0.56
Number of turns per phase	turn	564
Insulation thermal grade	°C	220

**Table 5:** The lifetime percentile of motor 1.

Lifetime Percentile	$B_{1}$	$B_{10}$	$B_{50}$
Lifetime/h	102,623	140,757	210,348

## Data Availability

The original contributions presented in the study are included in the article material, further inquiries can be directed to the corresponding authors.

## Abbreviations

The following abbreviations are used in this manuscript:

SCARA	Selective compliance assembly robot arm
PMSM	Permanent magnet synchronous motor
CFD	Computational fluid dynamics

## References

[1] Benotsmane R., Kovács G. (2023). Optimization of energy consumption of industrial robots using classical PID and MPC controllers. Energies.

[2] Arents J., Greitans M. (2022). Smart industrial robot control trends, challenges and opportunities within manufacturing. Appl. Sci..

[3] Liu L., Guo F., Zou Z., Duffy V.G. (2024). Application, development and future opportunities of collaborative robots (cobots) in manufacturing: A literature review. Int. J. Hum. Comput. Interact..

[4] Tang C., Yin X., Fang Y., Pfister P.D. (2023). A novel optimization framework for high dynamics point-to-point direct drive motion control system with a new type of surrogate model. IEEE Access.

[5] Zhao J., Wu C., Yang G., Chen C.Y., Chen S., Xiong C., Zhang C. (2022). Kinematics analysis and workspace optimization for a 4-DOF 3T1R parallel manipulator. Mech. Mach. Theory.

[6] Bu F., Xuan F., Yang Z., Gao Y., Pan Z., Degano M., Gerada C. (2020). Rotor position tracking control for low speed operation of direct-drive PMSM servo system. IEEE/ASME Trans. Mechatron..

[7] Kashiri N., Laffranchi M., Caldwell D.G., Tsagarakis N.G. (2015). Dynamics and control of an anthropomorphic compliant arm equipped with friction clutches. IEEE/ASME Trans. Mechatron..

[8] Dashti S., Ashourian M., Soheili A. (2020). Design and Implementation of Embedded Direct Drive SCARA Robot Controller with Resolved Motion Rate Control Method. Int. J. Adv. Des. Manuf. Technol..

[9] Henao H., Capolino G.A., Fernandez-Cabanas M., Filippetti F., Bruzzese C., Strangas E., Pusca R., Estima J., Riera-Guasp M., Hedayati-Kia S. (2014). Trends in fault diagnosis for electrical machines: A review of diagnostic techniques. IEEE Ind. Electron. Mag..

[10] International Electrotechnical Commission (2014). Rotating Electrical Machines-Part 18–41: Partial Discharge Free Electrical Insulation Systems (Type I) Used in Rotating Electrical Machines Fed from Voltage Converters—Qualification and Quality Control Tests.

[11] Ge X., Fan F., Given M.J., Stewart B.G. (2024). Insulation resistance degradation models of extruded power cables under thermal ageing. Energies.

[12] Dakin T.W. (1948). Electrical insulation deterioration treated as a chemical rate phenomenon. Trans. Am. Inst. Electr. Eng..

[13] Guo J., Li Z., Li M. (2019). A review on prognostics methods for engineering systems. IEEE Trans. Reliab..

[14] Stone G.C., Culbert I., Boulter E.A., Dhirani H. (2014). Electrical Insulation for Rotating Machines: Design, Evaluation, Aging, Testing, and Repair.

[15] Savin S., Ait-Amar S., Roger D. (2013). Turn-to-turn capacitance variations correlated to PDIV for AC motors monitoring. IEEE Trans. Dielectr. Electr. Insul..

[16] Ji Y., Giangrande P., Zhao W., Madonna V., Zhang H., Galea M. (2023). Determination of hotspot temperature margin for rectangular wire windings considering insulation thermal degradation and partial discharge. IEEE Trans. Transp. Electrif..

[17] Khowja M.R., Turabee G., Giangrande P., Madonna V., Cosma G., Vakil G., Gerada C., Galea M. (2021). Lifetime estimation of enameled wires under accelerated thermal aging using curve fitting methods. IEEE Access.

[18] Wang J., Xu L., Cai L., Zhang J., Tian J. CFD-based Temperature Field Analysis and Lifetime Prediction of Brushless DC Motor. Proceedings of the 2022 IEEE Transportation Electrification Conference and Expo, Asia-Pacific (ITEC Asia-Pacific).

[19] Turabee G., Khowja M.R., Madonna V., Giangrande P., Vakil G., Gerada C., Galea M. Thermal lifetime evaluation of electrical machines using neural network. Proceedings of the 2020 IEEE Transportation Electrification Conference & Expo (ITEC).

[20] Liu Z., Tang C., Fang Y., Pfister P.D. (2023). A Direct-Drive Permanent-Magnet Motor Selective Compliance Assembly Robot Arm: Modeling, Motion Control, and Trajectory Optimization Based on Direct Collocation Method. IEEE Access.

[21] Merat F. (1987). Introduction to robotics: Mechanics and control. IEEE J. Robot. Autom..

[22] He Y., Mai X., Cui C., Gao J., Yang Z., Zhang K., Chen X., Chen Y., Tang H. (2019). Dynamic modeling, simulation, and experimental verification of a wafer handling SCARA robot with decoupling servo control. IEEE Access.

[23] Wang J., Wu J., Zhang J., Zhang Q., Fang Y., Huang X. (2024). Remaining useful life prediction method by integrating two-phase accelerated degradation data and field information. IEEE Trans. Instrum. Meas..

[24] Liao H., Elsayed E.A. (2006). Reliability inference for field conditions from accelerated degradation testing. Nav. Res. Logist. (NRL).

[25] International Electrotechnical Commission (2011). Evaluation and Qualification of Electrical Insulation Systems.

[26] International Electrotechnical Commission (2014). Specification for Plastic Films for Electrical Purposes—Part 2: Methods of Test.

